# Connector Inversion Probe Technology: A Powerful One-Primer Multiplex DNA Amplification System for Numerous Scientific Applications

**DOI:** 10.1371/journal.pone.0000915

**Published:** 2007-09-19

**Authors:** Michael S. Akhras, Magnus Unemo, Sreedevi Thiyagarajan, Pål Nyrén, Ronald W. Davis, Andrew Z. Fire, Nader Pourmand

**Affiliations:** 1 Stanford Genome Technology Center, Stanford University, Palo Alto, California, United States of America; 2 Department of Biotechnology, Royal Institute of Technology, Stockholm, Sweden; 3 National Reference Laboratory for Pathogenic Neisseria, Department of Clinical Microbiology, Örebro University Hospital, Örebro, Sweden; 4 Department of Pathology, Stanford University School of Medicine, Stanford, California, United States of America; 5 Department of Genetics, Stanford University School of Medicine, Stanford, California, United States of America; 6 Biomolecular Engineering, University of California at Santa Cruz, Santa Cruz, California, United States of America; University of Liverpool, United Kingdom

## Abstract

We combined components of a previous assay referred to as Molecular Inversion Probe (MIP) with a complete gap filling strategy, creating a versatile powerful one-primer multiplex amplification system. As a proof-of-concept, this novel method, which employs a Connector Inversion Probe (CIPer), was tested as a genetic tool for pathogen diagnosis, typing, and antibiotic resistance screening with two distinct systems: i) a conserved sequence primer system for genotyping Human Papillomavirus (HPV), a cancer-associated viral agent and ii) screening for antibiotic resistance mutations in the bacterial pathogen *Neisseria gonorrhoeae*. We also discuss future applications and advances of the CIPer technology such as integration with digital amplification and next-generation sequencing methods. Furthermore, we introduce the concept of two-dimension informational barcodes, i.e. “multiplex multiplexing padlocks” (MMPs). For the readers' convenience, we also provide an on-line tutorial with user-interface software application CIP creator 1.0.1, for custom probe generation from virtually any new or established primer-pairs.

## Introduction

Since the introduction of PCR [1], a vast variety of offspring technologies have been engineered, continuously expanding our molecular toolbox [2]. One such technique was multiplex PCR [3], which allows for multiple reactions to occur in a single-tube. Multiplex PCR offers clear benefits in clinical management with respect to cost, time, sample need and contamination risk [4].

Recent advances in multiplex PCR technology have been significant [5]–[7]. Ligase Chain Reaction (LCR), an alternative method of DNA amplification, uses a thermostable ligase for target amplification [8]. In Gap-LCR [9], a thermostable polymerase is also used, and dNTPs are included to allow for gap filling of the flanking primers, mimicking nature's Okazaki fragments [10]. The padlock probe [11] introduced a novel concept of using a one-primer system for DNA target detection, based on ligase probe circularization and Rolling Circle Amplification (RCA) [12], [13]. The Molecular Inversion Probe (MIP) [14], a more advanced version of the padlock probe, contained also i) a molecular barcode for downstream chip detection, ii) a single base gap that enables single nucleotide polymorphism (SNP) detection, and iii) an exponential amplification strategy based on probe inversion and universal primer PCR amplification. MIP has since proved suitable as an ultra high-throughput method [15], showing potential for use in allele quantification [16] and pathogen diagnostics [17], [18].

**Figure 1:**
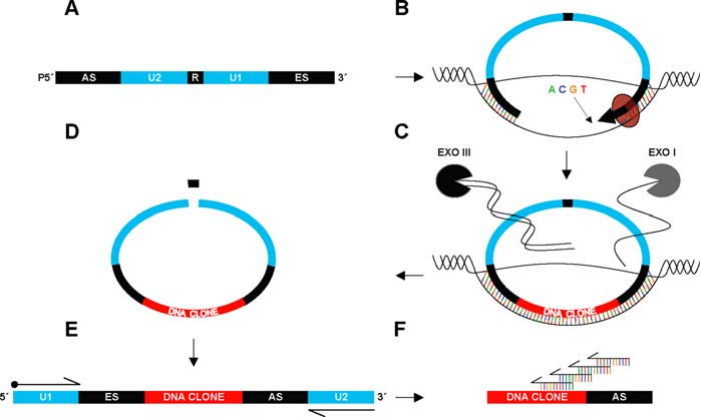
Schematic overview of Connector Inversion Probe (CIPer) technology. A) Synthetic oligonucleotide containing regions i) *AS and ES* (anchor site and extension sites): approximately 20 base pair fragments homologous to regions flanking target of interest, ii) *R*: restriction site for probe linearization, iii) *U1 and U2*: universal primer regions for inverted probe amplification. B) Target rescue through complete gap filling by a DNA polymerase and ligation-based probe circularization. C) Circular DNA enrichment through degradation of linear DNA by enzymes Exonuclease I and III, which are deactivated prior to the next step. D) Probe linearization by restriction site cleavage. E) Inverted probes are amplified with universal primers; one is biotinylated for subsequent amplicon validation. F) Amplicon validation using multiple sequencing primers.

We describe here a novel method, using a Connector Inversion Probe (CIPer), which combines the gap fill strategy of Gap-LCR with the structure of a padlock probe, creating a one-primer amplification with complete gap filling properties (Figure 1). PCR, a highly selective method, offers two degrees of selectivity, due to the use of two primers. CIPer technology uses only one primer, but retains two degrees of selectivity, owing to its dual detection; the target homologous extension site (ES) and anchor site (AS). CIPer also uses universal primer amplification in a one-protocol-fits-all fashion, which circumvents the need for primer-specific thermoprofiles. As a proof-of-concept, we tested CIPer on pathogen recognition with two distinct systems: i) conserved sequence primer genotyping of the cancer-associated viral agent Human Papillomavirus (HPV) [19], [20], and ii) a duplex amplification of two genes prone to mutations and strongly linked with resistance to key antibiotics (e.g. ciprofloxacin), in the bacterial pathogen *Neisseria gonorrhoeae* [21]–[23]. Previously developed Pyrosequencing protocols [24]–[26] were used for downstream sequence validation.

Here we introduce the design and development of CIPer as well as apply it with a robust protocol on viral and bacterial genetic diagnostics, typing and antibiotic resistance screening. We also provide an on-line tutorial with the user-interface software application CIP creator 1.0.1, for probe generation from virtually any primer pair (http://bioel.stanford.edu/CIPer).

## Results and Discussion

### Connector Inversion Probe (CIPer) Design and Performance

The Connector Inversion Probe (CIPer) reaction (Figure 1) shares some basic design features with a recently described assay, the PathogenMip assay [17], which includes: i) probe hybridization, single “G” base gap fill and probe circularization, ii) exonuclease based circular DNA selection, iii) probe inversion by re-linearization, and iv) universal PCR amplification. The distinguishing and key feature of the CIPer method is a modified first reaction step involving a complete filling of an extensive gap formed by the probe and target (Figure 1B).

The 3′end of the probe, which we call the Extension Site (ES), primes for polymerization, while the 5′end serves as point of ligation and is referred to as the Anchor Site (AS) (Figure 1). With all four dNTPs present, the polymerase is capable of extending past the intended point of ligation, generating a false negative. To address this problem, probes were designed to have an annealing temperature of approximately 5 degrees higher for the AS than that of the ES. The AS therefore blocks DNA extension when using a polymerase deficient in strand displacement at an appropriate intermediate temperature (50°C).

For enhanced sensitivity and specificity a vast variety of probe concentrations and thermoprofiles were explored. Probes used in the 10–100 fmol concentration range yielded the strongest signal. Higher cycle numbers and shorter annealing times created a more efficient thermoprofile. The CIPer method successfully genotyped 1 pg of plasmid DNA in the presence of 200 ng non-reacting human genomic DNA, without generating any false positives (Figure S1). Assay optimizations were based on genotyping of HPV plasmid 56, validated by either SYBR GREEN assay or agarose gel electrophoresis, followed by Pyrosequencing verification (see Results, *The Conserved Sequence Primer Connector Inversion Probe (CSP-CIPer*)).

### The Conserved Sequence Primer Connector Inversion Probe (CSP-CIPer)

The HPV CIPer (CSP-CIPer) was modeled on the primer-pair, GP5+/6+ [27], and exemplifies the use of conserved sequence primer (CSP) amplification. HPV genotyping is conventionally performed with a nested PCR [28]. The interest here lies in the detection potential of the CIPer compared with one-step PCR, and therefore the nested PCR strategy was not a suitable comparator for the model. The purpose of the CSP-CIPer was initially to genotype HPV plasmids -16 and -18, which produced amplicons visualized as agarose gel-bands at the expected size range of 185-188 base pairs and correctly validated with Pyrosequencing (Figure S2). With an accessible collection of 57 plasmids, a CSP-CIPer screen was performed in parallel with conventional GP5+/6+ PCR. All amplicons were validated as described in the Sentinel-base DNA genotyping method [25].

**Figure 2:**
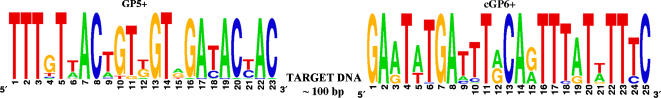
Sequence alignments (WebLogos, http://weblogo.berkeley.edu/logo.cgi) of the positive strand containing conserved sequence primer regions for GP5+/6+. The height of a DNA-base height represents the relative abundance of that particular base at the position of interest; multiple bases at the same position indicate a variable site. Alignment was performed for 40 genotypes detected with the CSP-CIPer or GP5+/6+ PCR (Table 1 and Figure S3). The target region between the two primers varies between 90-100 base pairs, depending on genotype characteristics. The positive strand of the GP6+ region is the complementary sequence of the established primer, and is therefore denoted cGP6+.

Among the non-detectable HPV genotypes were plasmids encoding HPV-6, -11, -27, -28, -34, -39, -40, -44 and -73. These genotypes all required a C-base instead of a T-base in the third position from the ES 3′end (Figure 2 and Figure S3). A degenerate CSP-CIPer was constructed with a mixed “Y” base (C+T) at the position of interest. The degenerate CSP-CIPer recognized and genotyped 35 plasmids, while the GP5+/6+ PCR genotyped 38 plasmids (Table 1). Successful target amplification schemes appear to be different for CIPers than for PCR primers; the success of a PCR primer-pair is highly dependent on the conservation of the 3'ends [29], while for the CIPer the anchor site seems more dependent upon annealing temperature. With the degenerate probe, detection rates improved by 15%. With proper mathematical modeling of aligned regions, higher detection success can be achieved with the use of degenerate bases or inosine residues [30].

**Table 1:** Genotyping data from CSP-CIPer and GP5+/6+ PCR parallel screens.

Method:	CIPer and PCR *(40 genotypes)*	CIPer only *(2 genotypes)*	PCR only *(5 genotypes)*	Undetected by either method *(17 genotypes)*
**HPV-type**	6b, 10, 11, 16, 18, 26, 27, 28, 29, 32, 33, 34, 35, 39, 40, 42, 44, 45, 52, 53, 54, 56, 58, 59, 62, 66, 67, 69, 70, 71, 72, 73, 74, 81, 82, 84, 86, 89, 90 and 91	27 and 29	28, 53, 71, 72 and 82	9, 12, 14, 15, 17, 19, 20, 21, 22, 23, 24, 36, 49, 50, 92, 93 and 96

HPV plasmids for 57 genotypes were used for testing and comparisons between the two systems. CSP-CIPer detected 35 plasmids, GP5+/6+ detected 38 plasmids, and 17 genotypes were not detected by either method. CSP-CIPer exclusively detected genotypes HPV-27 and -29, while GP5+/6+ PCR exclusively detected genotypes HPV-28, -53, -71, -72, and -82 (only HPV-53 is categorized as a high-risk genotype [20]).

To investigate the multiplexing strength of the CSP-CIPer, mixtures of 1-8 plasmids (encoding HPV-16, -18, -33, -35, -39, -45, -58 and -59) were made to mimic multiple co-infections, as these are known to occur. The GP5+/6+ primers could genotype four plasmids (encoding HPV-16, -18, -33 and -45) in the same mixture, while the CSP-CIPer could genotype all eight plasmids (Figure S4). The CSP-CIPer showed a 50% improved discriminatory power over that of the GP5+/6+ PCR. Differences in target amplification strategy account for this improvement. That is, PCR is based on target-dependent amplification, while the CIPer is based on target dependent probe circularization, followed by non-target dependent universal amplification (Figure 1).

### Antibiotic Resistance Mutation Screening

CIPer detection was also applied to screening of resistance mutations in *Neisseria gonorrhoeae*. Two CIPers were used to perform a duplex amplification in a single-tube of the genes *gyrA* and *parC* [23], which are known to mutate easily and by specific SNPs generate resistance to ciprofloxacin and other quinolone antibiotics. The CIPers were designed with CIP creator 1.0.1 (http://bioel.stanford.edu/CIPer) based on previously described PCR primer-pairs not originally intended for multiplex PCR [21]. We intentionally inverted the *gyrA* CIPer to target the negative strand, while the *parC* CIPer targeted the positive strand, to illustrate that CIPers can be used on both DNA strands. In the case of close target proximities, it might be wise to revise such a strategy. Mutations on amino acid residues S91 and D95 for *gyrA*, and E91 for *parC* were the particular subjects of our interrogation. There are many other candidate genes for future CIPer-based epidemiological studies of antibiotic resistance [31].

**Figure 3:**
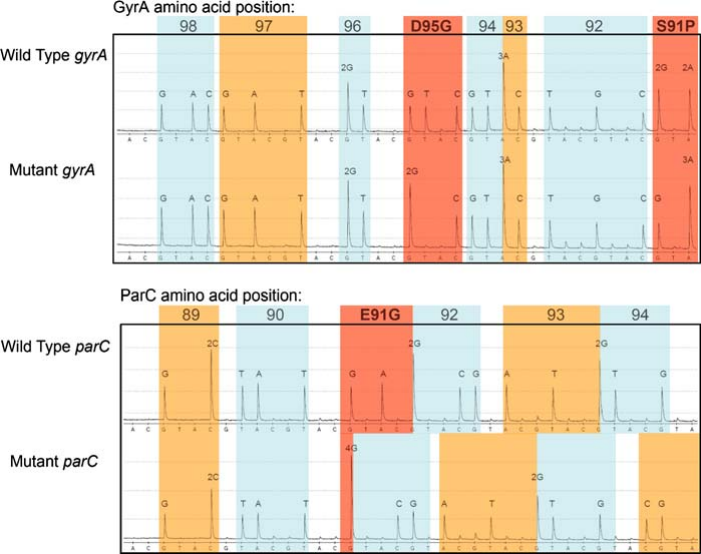
Pyrograms of Connector Inversion Probes (CIPer) used for screening of mutations conferring antibiotic (ciprofloxacin) resistance of *Neisseria gonorrhoeae* strains. Amino acid codons are marked alternating blue and orange, and their positions in the coded amino acid sequence of the target enzymes (GyrA and ParC) are placed on top of the traces. Codons marked in red are known to be subject to mutations, which are strongly correlated with ciprofloxacin resistance [21]–[23]. The *gyrA* and *parC* mutant sequences are compared with the respective wild type sequences. The displayed pyrograms show two common mutations (S91P and D95G) in *gyrA*, and one mutation (E91G) in *parC*.

Thirty-one *Neisseria gonorrhoeae* DNA extracts were screened in parallel with the CIPers in a single-tube reaction, and conventional PCR was performed in separate tubes for the two genes. Results from CIPer and PCR were in 100% agreement. In *gyrA*, eight wild type strains, three strains with double mutations resulting in amino acid alterations S91P and D95A, ten strains with double mutations resulting in S91P and D95G, eight strains with single mutation S91P, and two strains with single mutation D95N were observed. In *parC*, thirty wild type strains, and one strain with an E91G mutation was observed (Figure 3).

### Applications and Advances in Connector Inversion Probe (CIPer) Technology

Connector Inversion Probe (CIPer) technology is a powerful method for multiplex amplification, and has been validated here in genetic pathogen diagnostics, typing, and antibiotic resistance detection arenas as a proof-of-concept. This technology can be designed and optimized for use in numerous other scientific areas and applications. Other areas suitable for use of the CIPer method include cancer genomics [32], human SNP detection [15], and selective exon amplification [7], among numerous other promising applications. Modified CIPers may also in the future offer an alternative strategy for gateway cloning [33]. In this paper, we focused on “lowplex” systems amplifying shorter stretches of DNA of up to 100 base pair lengths. To achieve higher degrees of multiplexing and longer clone reads, one must take into account i) clone GC content, ii) clone length variability, and iii) steric effects for longer clones.

Analog amplification and downstream validation are delimiting factors for multiplex detection methods. We believe that the future of CIPer technology will rely on digital amplification [34] and high throughput sequencing validation (Figure S5). The CIPers universal primer sites can be formatted to fit any amplification method, e.g. RCA [12], [13], emulsion PCR [34], SPIA [35] LAMP [36] and SMAP 2 [37]; universal adaptor ligation, a tedious and sample-consuming procedure [38], is therefore optional. Proposed digital amplification methods include emulsion PCR, RCA, or a combination of the two [39]. Suitable upcoming high throughput validation methods include next-generation sequence systems such as the 454 Life Sciences platform [38] (http://www.454.com), the Solexa platform [40] (http://www.illumina.com), polony sequencing [41] (http://www.agentcourt.com), Helicos Biosciences [42] (http://www.helicosbio.com), or use of a resequencing array (GeneChip CustomSeq, www.affymetrix.com). Recent digital quantification methods [43] open the possibility for quantitative CIPer technology.

With the addition of a molecular barcode to the padlocks [14], MIPs were used for ultra high-throughput SNP detection based on microarray barcode hybridization [15]. Because CIPers can also include a barcode, they could be used for microarray validation, or amplicon tracing with sequencing methods [17], [44] allowing for longer read lengths [38]. With the introduction of a third universal sequence flanking the barcode and the universal primer sites, one can begin to envision a two-dimension informational barcode, a concept we call “multiplexing multiplex padlocks” (MMP) (Figure S6). Probe construction based on ligation of three smaller constructs [45] would allow for variability in fragment assembly. By combining the one-genotype detection pair of AS/ES constructs with multiple unique barcodes, we can create patient and genotype specific probes with one barcode per probe. This will allow for analysis of multiple parallel samples, essentially lowering the cost of microarray chips and sequencing reactions.

## Materials and Methods

### DNA Materials

Oligonucleotides used in the assay (Table S1) were produced by IDT technologies (Coralville, IA) and melting temperatures were calculated using SciTools (IDT technologies). HPV plasmids were kindly provided by Dr. E. M. de Villiers (DKFZ; Heidelberg, Germany) HPV-6B, -11, -16, -18, -26, -27, -40, -45, -53, -72 and -73; HPV-9, -10, -12, -14, -15, -17, -19, -20, -21, -22, -23, -24, -28, -29, -32, -33, -34, -36, -39, -42, -49, -50, -54, -66, -70 and -74 by Dr. M. Favre (Institute Pasteur; Paris, France); HPV-35, -44 and -56 by Dr. A. Lorincz (Digene Corporation; Gaithersburg, MD); HPV-58, -59, -67, -69, -71, -81 and -82 by Dr. T. Matsukura (National Institute of Health; Tokyo, Japan); HPV-52 by Dr. W. Lancaster (Wayne State University School of Medicine; Detroit, MI); HPV-62, -84, -cand86, -cand89, -90 and -91 by Dr. R. D. Burk (Albert Einstein College of Medicine of Yeshiva University, Bronx, NY); HPV-92, -93 and -96 by Dr. O. Forslund (UMAS; Malmö University Hospital, Malmö, Sweden). All HPV plasmids were normalized at 100 ng/µl (±15%) using a ND-100 Spectrophotometer (NanoDrop, Wilmington, DE). *Neisseria gonorrhoeae* DNA had been previously extracted [24]. Commercially available human genomic DNA G3041 (Promega, Madison, WI) was used for assay evaluation.

### Connector Inversion Probe Assay

CIPer reactions were performed as follows: **i)** 100 ng of HPV plasmid DNA or *Neisseria gonorrhoeae* DNA extract, 10 fmol each of probe, 0.25 units Ampligase (Epicenter Biotechnologies, Madison, WI), 0.25 mM of each dNTP (Fermentas, Hanover, MD), and 0.5 units AmpliTaq DNA Polymerase Stoffel Fragment (Applied Biosystems, Foster City, CA) in 1X Ampligase buffer (Epicenter Biotechnologies, Madison, WI) were combined in a total volume of 10 µl/reaction. After pre-incubation at 95°C for 10 min, the reaction went through 99 cycles of 95°C for 2 min, 50°C for 5 min and 37°C for 2 min. **ii)** 10 µl of 130 mM Tris-HCl (pH 7.8), 85.75 mM KCl, 3.85 mM MgCl_2_ and 0.1% BSA solution containing 5.6 units Exonuclease I and 112 units Exonuclease III (Epicenter Biotechnologies, Madison, WI) was added to each reaction. The mixture was incubated at 37°C for 60 min followed by enzyme deactivation at 80°C for 20 min. **iii)** 4 units of Uracil-DNA-Glycosylase (New England BioLabs, Ipswich, MA) was added to each reaction and incubated at 37°C for 60 min followed by enzyme deactivation at 80°C for 20 min. **iv)** PCR amplification was carried out in a total reaction volume of 50 µl, containing 5 µl CIPer reaction, 1X PCR Buffer II (Applied Biosystems, Foster City, CA), 2.5 mM MgCl_2_ (Applied Biosystems, Foster City, CA), 0.12 mM dNTPs (Fermentas, Hanover, MD), 2.5 units AmpliTaq Gold DNA polymerase (Applied Biosystems, Foster City, CA) and 0.2 µM of each universal primer B-NP-F and NP-R (Table S1). A 10-min incubation step at 95°C was followed by 40 cycles of amplification with a thermocycler GeneAmp PCR system 9700 (Applied Biosystems, Foster City, CA). Each cycle included a denaturation step at 95°C for 45 sec, an annealing step at 52°C for 30 sec, and an extension step at 72°C for 30 sec. A final extension was done at 72°C for 5 min. Real-time PCR reactions were performed with the same thermoprofile on an Mx3005P system (Stratagene, La Jolla, CA), with a SYBR green PCR master mix (Applied Biosystems, Foster City, CA). Gel electrophoresis was performed on a Sub-Cell Gt Agarose Electrophoresis System (Bio-Rad, Hercules, CA) using a 2.5% agarose gel GenePure LE Quick Dissolve (ISC Bioexpress, Kaysville, UT) with 5 µg/ml ethidium bromide (Sigma-Aldrich, St. Louis, MO) staining.

### Pyrosequencing

Single strand template preparation was performed as previously described [26]. GP5+ and Sentinel-base sequencing primers were used to screen all HPV CSP-CIPer clones and GYRA2-4 and PARC-3 sequencing primers were used for *Neisseria gonorrhoeae* CIPer clones (Table S1). Pyrosequencing was performed with a cyclic *de novo* sequencing dispensation (ACGT) using a PSQ™ HS96A DNA sequencing system (Biotage, Uppsala, Sweden).

## Supporting Information

Figure S1 Agarose gel stained with ethidium bromide for CSP-CIPer detection limit of HPV -56 in presence of human genomic DNA. The plasmid concentration was varied, ranging from 10 ng to 100 fg per reaction at a constant background of 200 ng non-HPV-contaminated human genomic DNA. The minimum detectable amount of HPV observed was 1 pg, and the 100 fg mixture showed no significant amplification. The upper triangular graph represents a visual interpretation of CIPer amplicon intensity as a function of plasmid concentration, and the lower graph the background intensity as a function of plasmid concentration.
(0.06 MB PDF)

Figure S2 Agarose gel stained with ethidium bromide for fragment size determination and pyrograms for HPV genotypes -16 and -18. Amplicon sizes appear in the expected size of 185 base pairs for HPV-16 and 188 base pairs for HPV-18. The negative controls for the CIPer reaction (“CIPer-”) and PCR (“PCR-”) show no significant amplification. Pyrograms derived from multiple sequencing primers MSP-16 and MSP-18 (Table S1) validate the expected sequence.
(0.21 MB PDF)

Figure S3 Genomic regions GP5+/6+ aligned for 40 genotypes detected by either CSP-CIPer or GP5+/6+ PCR (Table 1). Alignment was performed and displayed with ClustalX (http://www.biodirectory.com/biowiki/ClustalX). The targeted region flanking the two primers varies between 90-100 base pairs depending on genotype characteristics, and is represented in the figure with three Ns.
(0.30 MB PDF)

Figure S4 Detecting multiple HPV co-infections. In order to compare the discriminative power of the CIPer vs. PCR detection, artificial mixtures of the eight plasmids HPV-16, -18, -33, -35, -39, -45, -58 and -59 were constructed to “mimic” real-case multiple co-infections. The CIPer method could detect all eight genotypes present in the same sample, while PCR managed with the four genotypes HPV-16, -18, -33 and -45. As seen in the figure the Pyrogram intensities vary for different genotypes, indicating a lower discriminative preference for certain types. Among the four PCR detected genotypes, HPV-16 and -33 showed very weak diagrams and were barely detectable in the presence of the preferred genotypes -18 and -45. CIPer detection suffered to a lesser degree of such preferred selectivity, as all types were clearly distinguishable. We believe that differences in target amplification strategy account for this improvement, i.e. PCR is based on target dependent amplification, while the CIPer is based on target dependent probe circularization, followed by non-target dependent universal amplification.
(0.64 MB PDF)

Figure S5 Digital amplification strategies prior to probes validation. A) Emulsion based PCR; adapters are optional since CIPers already contain universal segments flanking the target of interest. The technology involves the inclusion of DNA and a primed magnetic bead in mineral oil (an emulsion), which allows for single molecule amplifications. Suitable upcoming methods for downstream sequence validation include the 454 Life Sciences platform (http://www.454.com), the Solexa platform (http://www.illumina.com), polony sequencing (http://www.agentcourt.com), Helicos Biosciences (http://www.helicosbio.com), or use of a resequencing array (GeneChip CustomSeq, www.affymetrix.com). B) Rolling circle amplification (RCA) with single-molecule detection (SMD). Digital quantification combines RCA and SMD in form of fluorescent-labeled target specific oligonucleotides. The amplified CIPers can be quantified using microfluidic analysis and visualized with a microscope for ultimate levels of quantification. The number of available fluorescent labels limits the degree of multiplexing.
(0.06 MB PDF)

Figure S6 “Multiplex multiplexing padlocks” (MMP) construct assembly for CIPer probes production. The extended CIPer carries a third universal segment and flanks the barcode together with the other universal segments. The probe is divided and synthesized in three constructs, with the middle one containing the barcode. Through a two-way ligation scheme the three fragments can be joined into a full-length probe. Following the grid-like assembly strategy displayed in the figure, one-genotype detection pair of AS/ES constructs is combined with multiple unique barcodes. The unique barcode now carries two dimensions of information, i.e. genotype and patient ID. Post-reaction CIPers can be combined into multiple patient pools and all screened simultaneously, essentially lowering costs involved with downstream validations, both for hybridization-based techniques and sequencing procedures.
(0.05 MB PDF)

Table S1 Oligonucleotides used in the present study. Bold italic marked sequences in the anchor sites of CIPers -gyrA and +parC were added to the originally described primers [21] to obtain higher annealing temperature values for the anchor site. The number in parenthesis following the multiple sequencing primers (MSPs) denotes which primer pool the MSP belongs to.
(0.09 MB PDF)
