# Supplementary material for: Connector Inversion Probe Technology: A Powerful One-Primer Multiplex DNA Amplification System for Numerous Scientific Applications
Source: PLoS One. 2007 Sep 19;2(9):e915. doi: 10.1371/journal.pone.0000915 (PMC1976392; doi:10.1371/journal.pone.0000915)
Supplement: Figure S1 — Agarose gel stained with ethidium bromide for CSP-CIPer detection limit of HPV -56 in presence of human genomic DNA. The plasmid concentration was varied, ranging from 10 ng to 100 fg per reaction at a constant background of 200 ng non-HPV-contaminated human genomic DNA. The minimum detectable amount of HPV observed was 1 pg, and the 100 fg mixture showed no significant amplification. The upper triangular graph represents a visual interpretation of CIPer amplicon intensity as a function of plasmid concentration, and the lower graph the background intensity as a function of plasmid concentration. (0.06 MB PDF) [file pone.0000915.s001.pdf]

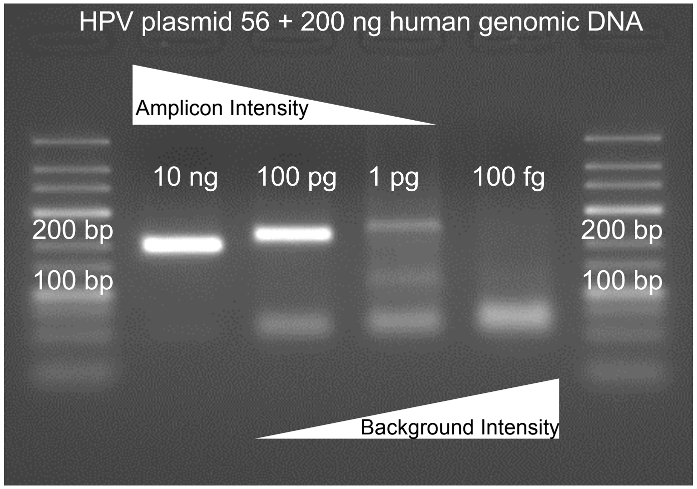

Figure S1. Agarose gel stained with ethidium bromide for CSP-CIPer detection limit of HPV -56 in presence of human genomic DNA. The plasmid concentration was varied, ranging from 10 ng to 100 fg per reaction at a constant background of 200 ng non-HPV-contaminated human genomic DNA. The minimum detectable amount of HPV observed was 1 pg, and the 100 fg mixture showed no significant amplification. The upper triangular graph represents a visual interpretation of CIPer amplicon intensity as a function of plasmid concentration, and the lower graph the background intensity as a function of plasmid concentration.
