# Supplementary material for: Connector Inversion Probe Technology: A Powerful One-Primer Multiplex DNA Amplification System for Numerous Scientific Applications
Source: PLoS One. 2007 Sep 19;2(9):e915. doi: 10.1371/journal.pone.0000915 (PMC1976392; doi:10.1371/journal.pone.0000915)
Supplement: Figure S3 — Genomic regions GP5+/6+ aligned for 40 genotypes detected by either CSP-CIPer or GP5+/6+ PCR (Table 1). Alignment was performed and displayed with ClustalX (http://www.biodirectory.com/biowiki/ClustalX). The targeted region flanking the two primers varies between 90-100 base pairs depending on genotype characteristics, and is represented in the figure with three Ns. (0.30 MB PDF) [file pone.0000915.s003.pdf]

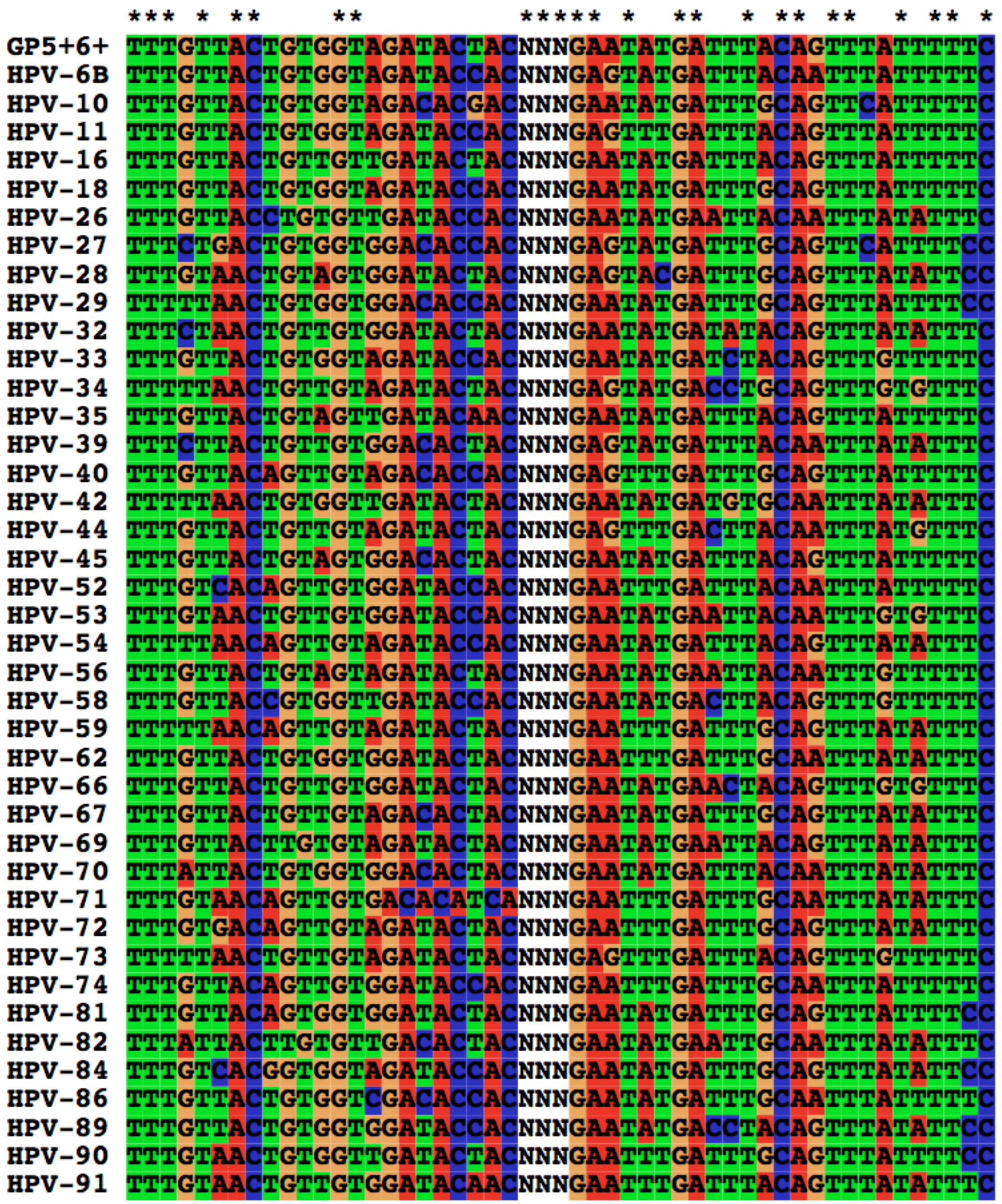

Figure S3. Genomic regions GP5+/6+ aligned for 40 genotypes detected by either CSP-CIPer or GP5+/6+ PCR (Table 1). Alignment was performed and displayed with ClustalX (http://www.biodirectory.com/biowiki/ClustalX). The targeted region flanking the two primers varies between 90-100 base pairs depending on genotype characteristics, and is represented in the figure with three Ns.
