# Supplementary material for: Connector Inversion Probe Technology: A Powerful One-Primer Multiplex DNA Amplification System for Numerous Scientific Applications
Source: PLoS One. 2007 Sep 19;2(9):e915. doi: 10.1371/journal.pone.0000915 (PMC1976392; doi:10.1371/journal.pone.0000915)
Supplement: Figure S4 — Detecting multiple HPV co-infections. In order to compare the discriminative power of the CIPer vs. PCR detection, artificial mixtures of the eight plasmids HPV-16, -18, -33, -35, -39, -45, -58 and -59 were constructed to “mimic” real-case multiple co-infections. The CIPer method could detect all eight genotypes present in the same sample, while PCR managed with the four genotypes HPV-16, -18, -33 and -45. As seen in the figure the Pyrogram intensities vary for different genotypes, indicating a lower discriminative preference for certain types. Among the four PCR detected genotypes, HPV-16 and -33 showed very weak diagrams and were barely detectable in the presence of the preferred genotypes -18 and -45. CIPer detection suffered to a lesser degree of such preferred selectivity, as all types were clearly distinguishable. We believe that differences in target amplification strategy account for this improvement, i.e. PCR is based on target dependent amplification, while the CIPer is based on target dependent probe circularization, followed by non-target dependent universal amplification. (0.64 MB PDF) [file pone.0000915.s004.pdf]

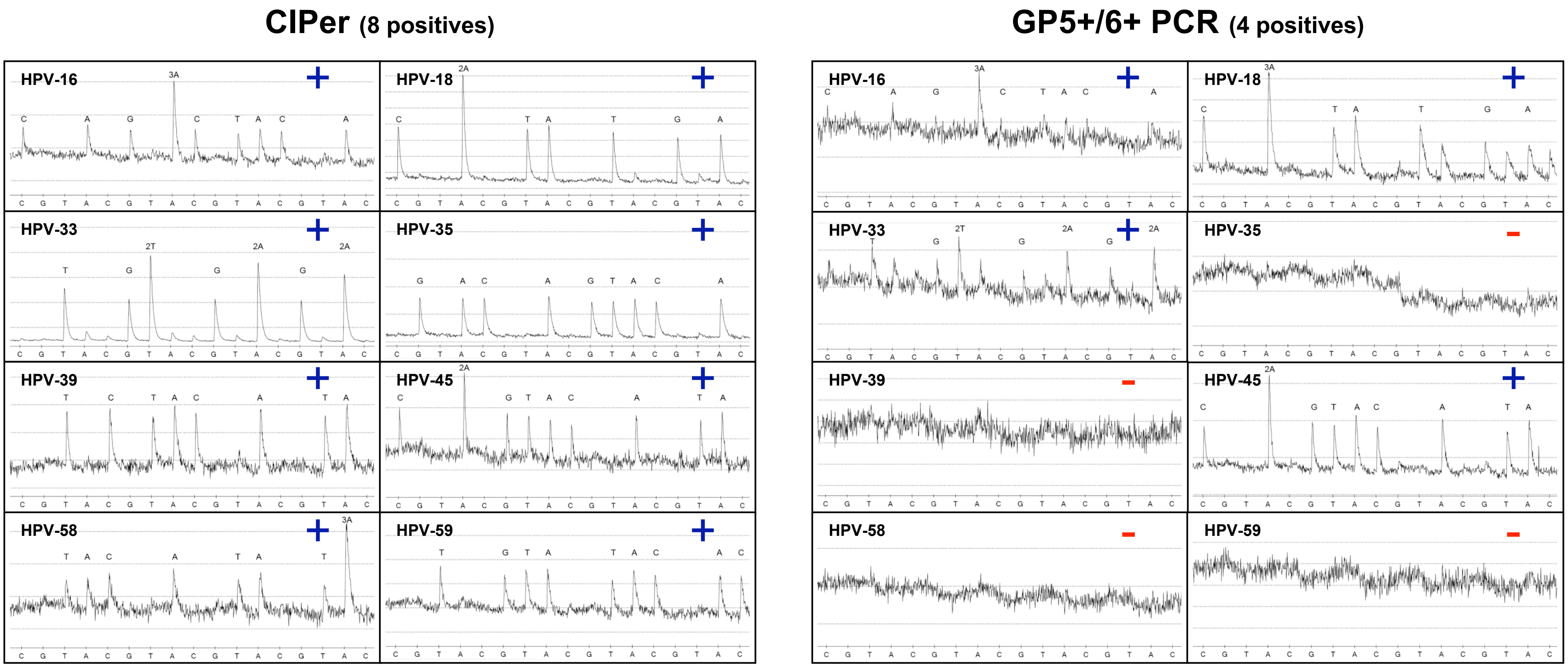

Figure S4. Detecting multiple HPV co-infections. In order to compare the discriminative power of the CIPer vs. PCR detection, artificial mixtures of the eight plasmids HPV-16, -18, -33, -35, -39, -45, -58 and -59 were constructed to “mimic” real-case multiple co-infections. The CIPer method could detect all eight genotypes present in the same sample, while PCR managed with the four genotypes HPV-16, -18, -33 and -45. As seen in the figure the Pyrogram intensities vary for different genotypes, indicating a lower discriminative preference for certain types. Among the four PCR detected genotypes, HPV-16 and -33 showed very weak diagrams and were barely detectable in the presence of the preferred genotypes -18 and -45. CIPer detection suffered to a lesser degree of such preferred selectivity, as all types were clearly distinguishable. We believe that differences in target amplification strategy account for this improvement, i.e. PCR is based on target dependent amplification, while the CIPer is based on target dependent probe circularization, followed by non-target dependent universal amplification.
