# Supplementary material for: Connector Inversion Probe Technology: A Powerful One-Primer Multiplex DNA Amplification System for Numerous Scientific Applications
Source: PLoS One. 2007 Sep 19;2(9):e915. doi: 10.1371/journal.pone.0000915 (PMC1976392; doi:10.1371/journal.pone.0000915)
Supplement: Figure S5 — Digital amplification strategies prior to probes validation. A) Emulsion based PCR; adapters are optional since CIPers already contain universal segments flanking the target of interest. The technology involves the inclusion of DNA and a primed magnetic bead in mineral oil (an emulsion), which allows for single molecule amplifications. Suitable upcoming methods for downstream sequence validation include the 454 Life Sciences platform (http://www.454.com), the Solexa platform (http://www.illumina.com), polony sequencing (http://www.agentcourt.com), Helicos Biosciences (http://www.helicosbio.com), or use of a resequencing array (GeneChip CustomSeq, www.affymetrix.com). B) Rolling circle amplification (RCA) with single-molecule detection (SMD). Digital quantification combines RCA and SMD in form of fluorescent-labeled target specific oligonucleotides. The amplified CIPers can be quantified using microfluidic analysis and visualized with a microscope for ultimate levels of quantification. The number of available fluorescent labels limits the degree of multiplexing. (0.06 MB PDF) [file pone.0000915.s005.pdf]

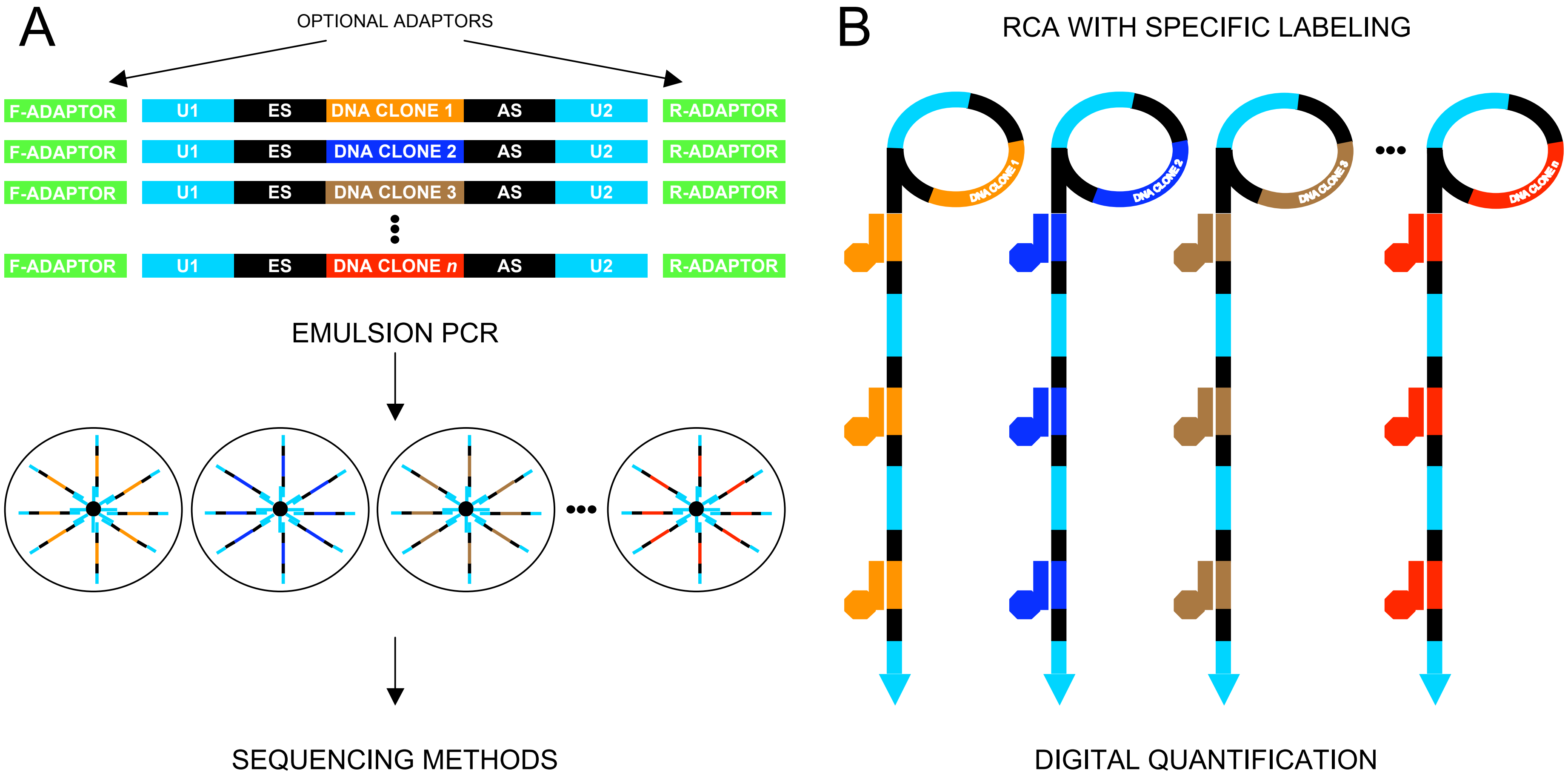

Figure S5. Digital amplification strategies prior to probes validation. A) Emulsion based PCR; adapters are optional since CIPers already contain universal segments flanking the target of interest. The technology involves the inclusion of DNA and a primed magnetic bead in mineral oil (an emulsion), which allows for single molecule amplifications. Suitable upcoming methods for downstream sequence validation include the 454 Life Sciences platform (http://www.454.com), the Solexa platform (http://www.illumina.com), polony sequencing (http://www.agentcourt.com), Helicos Biosciences (http://www.helicosbio.com), or use of a resequencing array (GeneChip CustomSeq, www.affymetrix.com). B) Rolling circle amplification (RCA) with single-molecule detection (SMD). Digital quantification combines RCA and SMD in form of fluorescent-labeled target specific oligonucleotides. The amplified CIPers can be quantified using microfluidic analysis and visualized with a microscope for ultimate levels of quantification. The number of available fluorescent labels limits the degree of multiplexing.
