# Supplementary material for: Connector Inversion Probe Technology: A Powerful One-Primer Multiplex DNA Amplification System for Numerous Scientific Applications
Source: PLoS One. 2007 Sep 19;2(9):e915. doi: 10.1371/journal.pone.0000915 (PMC1976392; doi:10.1371/journal.pone.0000915)
Supplement: Figure S6 — “Multiplex multiplexing padlocks” (MMP) construct assembly for CIPer probes production. The extended CIPer carries a third universal segment and flanks the barcode together with the other universal segments. The probe is divided and synthesized in three constructs, with the middle one containing the barcode. Through a two-way ligation scheme the three fragments can be joined into a full-length probe. Following the grid-like assembly strategy displayed in the figure, one-genotype detection pair of AS/ES constructs is combined with multiple unique barcodes. The unique barcode now carries two dimensions of information, i.e. genotype and patient ID. Post-reaction CIPers can be combined into multiple patient pools and all screened simultaneously, essentially lowering costs involved with downstream validations, both for hybridization-based techniques and sequencing procedures. (0.05 MB PDF) [file pone.0000915.s006.pdf]

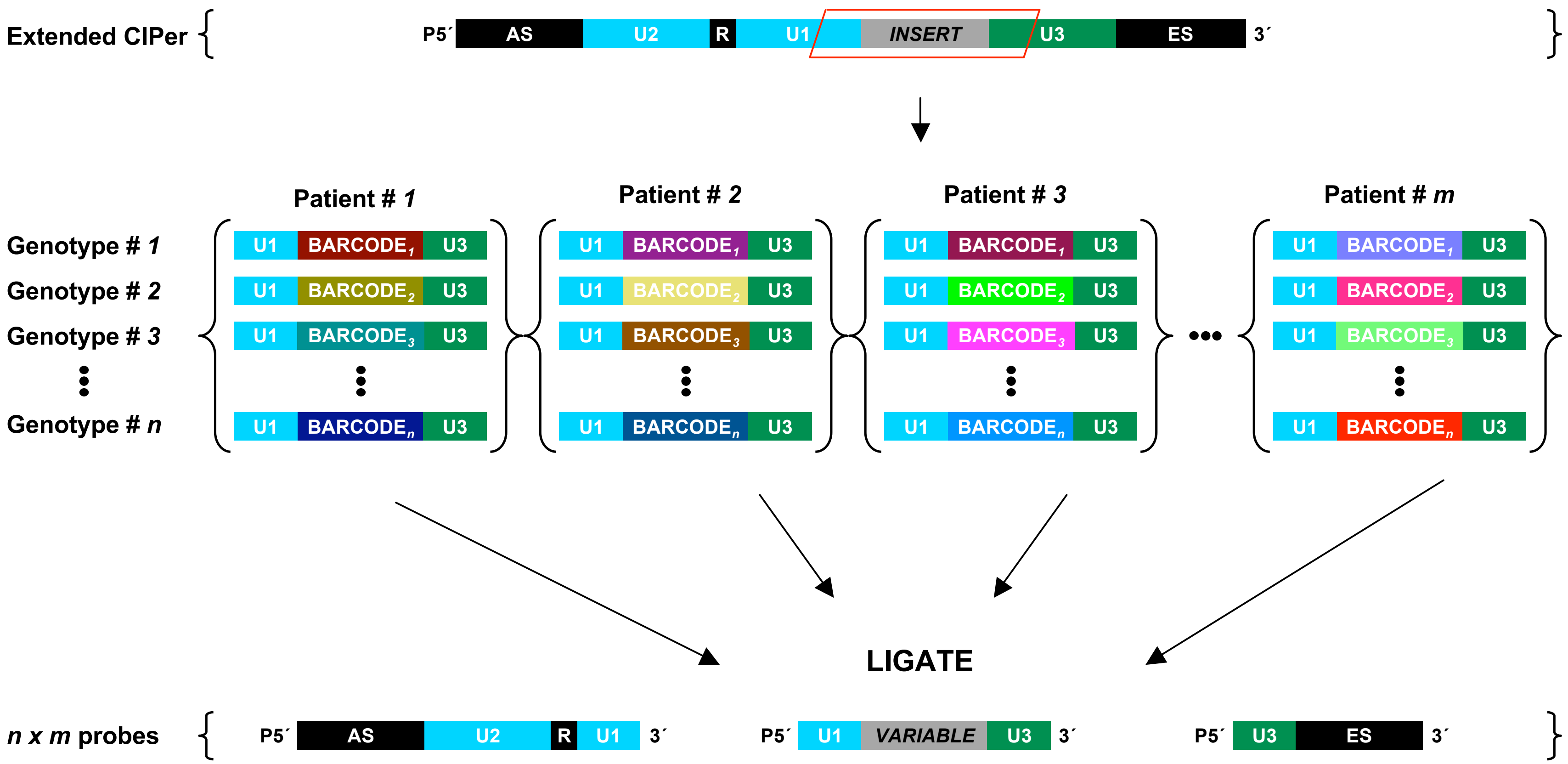

Figure S6. "Multiplex multiplexing padlocks" (MMP) construct assembly for CIPer probes production. The extended CIPer carries a third universal segment and flanks the barcode together with the other universal segments. The probe is divided and synthesized in three constructs, with the middle one containing the barcode. Through a two-way ligation scheme the three fragments can be joined into a full-length probe. Following the grid-like assembly strategy displayed in the figure, one-genotype detection pair of AS/ES constructs is combined with multiple unique barcodes. The unique barcode now carries two dimensions of information, i.e. genotype and patient ID. Post-reaction CIPers can be combined into multiple patient pools and all screened simultaneously, essentially lowering costs involved with downstream validations, both for hybridization-based techniques and sequencing procedures.
