# Supplementary material for: Connector Inversion Probe Technology: A Powerful One-Primer Multiplex DNA Amplification System for Numerous Scientific Applications
Source: PLoS One. 2007 Sep 19;2(9):e915. doi: 10.1371/journal.pone.0000915 (PMC1976392; doi:10.1371/journal.pone.0000915)
Supplement: Table S1 — Oligonucleotides used in the present study. Bold italic marked sequences in the anchor sites of CIPers -gyrA and +parC were added to the originally described primers [21] to obtain higher annealing temperature values for the anchor site. The number in parenthesis following the multiple sequencing primers (MSPs) denotes which primer pool the MSP belongs to. (0.09 MB PDF) [file pone.0000915.s007.pdf]

**A. CIPer**	**/5Phos/------------AS--U2----------------R----------------U1---ES-------------------------3´**		
CSP-CIPer	GTAGTATCTACCACAGTAACAAA	CATACCTACACTAACTTCTA**U**U**U**ACACATTACTCACCCTATTA	GAAAAATAAACTGTAAATCATAYTC
- *gyrA*	TGTGCTGATAGACGGACAGGG***CAA C***	CATACCTACACTAACTTCTA**U**U**U**ACACATTACTCACCCTATTA	TAACTGGAATGCCGCCTACAA
+*parC*	CCAAAATCTCGCCGACCACGC***GCGC***	CATACCTACACTAACTTCTA**U**U**U**ACACATTACTCACCCTATTA	GCGAACCGAAGTTGCCGATGC
B. PCR	5´-3´		
B-F-NP	/5Bio/ACACATTACTCACCCTATTA		
R-NP	TAGAAGTTAGTGTAGGTATG		
GP5+	TTTGTTACTGTGGTAGATACTAC		
GP6+	5/Bio/GAAAAATAAACTGTAAATCATATTC		
GYRA2-1	TAACTGGAATGCCGCCTACAA		
GYRA2-2	/5Bio/CCCTGTCCGTCTATCAGCACA		
PARC-1	GCGTGGTCGGCGAGATTTTGG		
PARC-2	/5Bio/GCGAACCGAAGTTGCCGATGC		
C. Sequencing	5´-3´		
GP5+	TTTGTTACTGTGGTAGATACTAC		
MSP-16 (1)	ATTATGTGCTGCCATATCTACTT		
MSP-31 (1)	GTGCTGCAATTGCAAACAGT		
MSP-59 (1)	CTACTACTTCTTCTATTCCTAA		
MSP-39 (1)	CTATAGAGTCTTCCATACCT		
MSP-18 (2)	ACAGTCTCCTGTACCTGGG		
MSP-33 (2)	TGAGAATTTTAAAGAATATATAAGACA		
MSP-52 (2)	TATGTGCTGAGGTTAAAAAG		
MSP-56 (2)	TACTAACATGACTATTAGTACT		
MSP-45 (3)	CTCTACACAAAATCCTGTGC		
MSP-35 (3)	GTTCTGCTGTGTCTTCTAGT		
MSP-58 (3)	ACTGAAGTAACTAAGGAAGG		
MSP-51 (3)	ACTATTAGCACTGCCACTGC		
GYRA2-4	AGCGAAATTTTGCGCCATACG		
PARC-3	ATCCGCACGGCGACAGTTCC		

Figure S7. Oligonucleotides used in the present study. Bold italic marked sequences in the anchor sites of CIPers -*gyrA* and +*parC* were added to the originally described primers to obtain higher annealing temperature values for the anchor site. The number in parenthesis following the multiple sequencing primers (MSPs) denotes which primer pool the MSP belongs to.
