# Supplementary material for: Connector Inversion Probe Technology: A Powerful One-Primer Multiplex DNA Amplification System for Numerous Scientific Applications
Source: PLoS One. 2007 Sep 19;2(9):e915. doi: 10.1371/journal.pone.0000915 (PMC1976392; doi:10.1371/journal.pone.0000915)
Supplement: Figure S2 — Agarose gel stained with ethidium bromide for fragment size determination and pyrograms for HPV genotypes -16 and -18. Amplicon sizes appear in the expected size of 185 base pairs for HPV-16 and 188 base pairs for HPV-18. The negative controls for the CIPer reaction (“CIPer-”) and PCR (“PCR-”) show no significant amplification. Pyrograms derived from multiple sequencing primers MSP-16 and MSP-18 (Table S1) validate the expected sequence. (0.21 MB PDF) [file pone.0000915.s002.pdf]

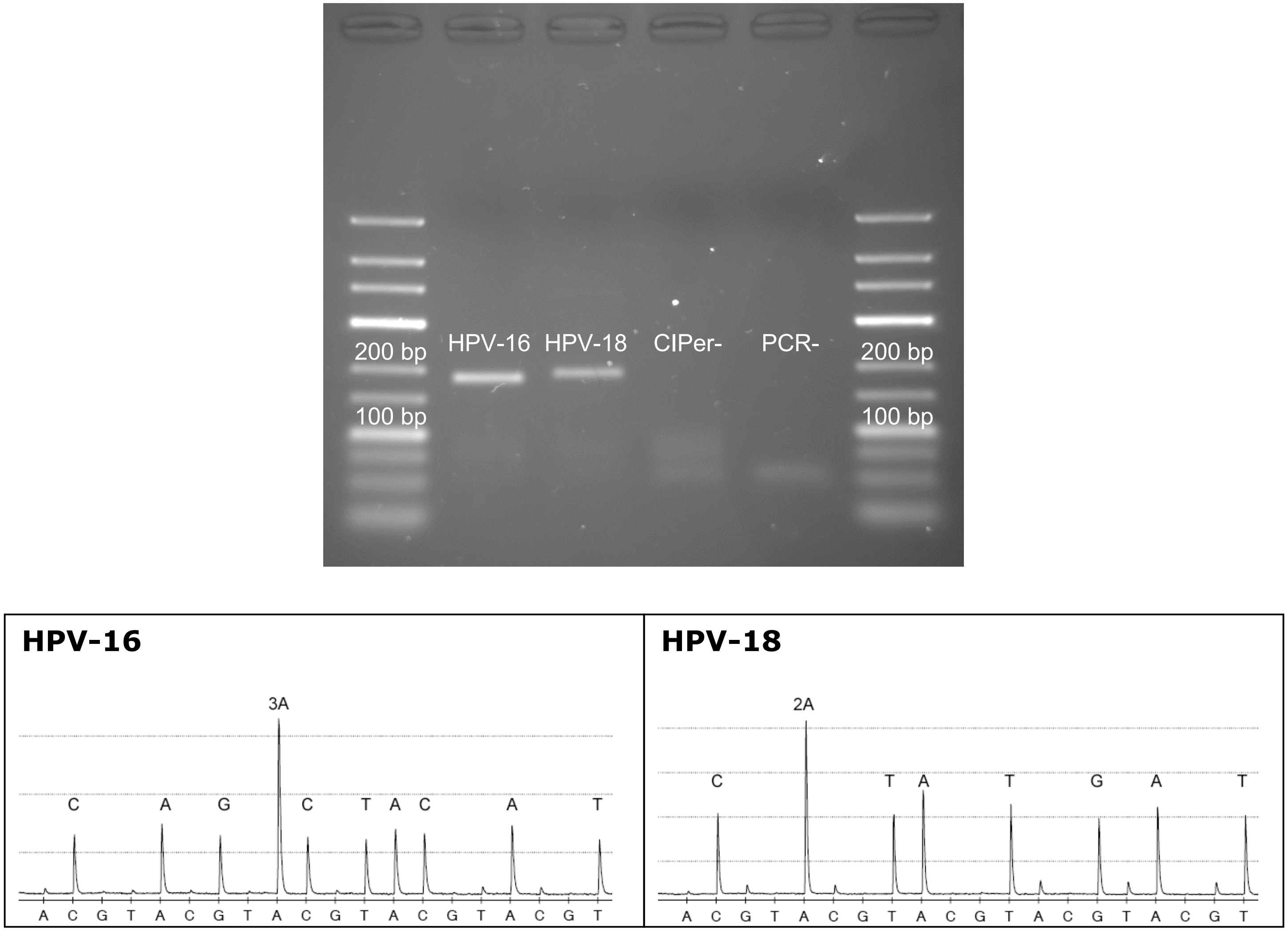

Figure S2. Agarose gel stained with ethidium bromide for fragment size determination and pyrograms for HPV genotypes -16 and -18. Amplicon sizes appear in the expected size of 185 base pairs for HPV-16 and 188 base pairs for HPV-18. The negative controls for the CIPer reaction ("CIPer-") and PCR ("PCR-") show no significant amplification. Pyrograms derived from multiple sequencing primers MSP-16 and MSP-18 (Table S1) validate the expected sequence.
